# Impact of Bristle Hardness of Manual Toothbrushes on the Cleaning Efficacy of Teeth with Fixed Orthodontic Appliances

**DOI:** 10.3290/j.ohpd.c_2638

**Published:** 2026-04-13

**Authors:** Damian Wüthrich, Andrea Gubler, Thomas Attin, Florian J. Wegehaupt

**Affiliations:** a Damian Wüthrich Postgraduate Student, Clinic of Conservative and Preventive Dentistry, Center for Dental Medicine, University of Zürich, Zürich, Switzerland. Research idea, experimental design, wrote the manuscript, performed the experiments in fulfilment of requirements for a doctoral degree.; b Andrea Gubler Head of Research Laboratory, Clinic of Conservative and Preventive Dentistry, Center for Dental Medicine, University of Zürich, Zürich, Switzerland. Supported and organized the implementation of the experiments.; c Thomas Attin Professor and Director, Clinic of Conservative and Preventive Dentistry, Center for Dental Medicine, University of Zürich, Zürich, Switzerland. Contributed substantially to concept, discussion, proofread the manuscript.; d Florian J. Wegehaupt Professor and Head Division of Preventive Dentistry and Oral Epidemiology, Clinic of Conservative and Preventive Dentistry, Center for Dental Medicine, University of Zürich, Zürich, Switzerland. Research idea, hypothesis, experimental design, contributed substantially to discussion and writing the paper, proofread the manuscript.

**Keywords:** cleaning performance, oral hygiene, orthodontic appliances, toothbrush wear.

## Abstract

**Purpose:**

To evaluate the influence of bristle hardness (soft or medium) and simulated aging of manual toothbrushes on the cleaning efficacy in teeth with fixed orthodontic appliances.

**Materials and Methods:**

Brackets were attached to black resin teeth coated with a layer of titanium dioxide and brushed using a soft and medium toothbrush. Two different brushing motions, horizontal and circular, were tested. After every brushing session, the percentage of cleaned areas was measured to evaluate the cleaning performance. Brushing with the respective toothbrushes on a 3D-printed typodont with brackets and wire was performed to simulate the aging of the brush. Cleaning performance was reassessed after simulated periods of two, four and six months. Statistical analysis was performed at baseline and after six months of simulated use, using beta regression and pairwise comparisons.

**Results:**

Toothbrushes with softer bristles consistently cleaned more effectively than harder ones, regardless of brushing motion (p < 0.0001, respectively). At all timepoints, horizontal brushing was also more effective than circular brushing. The cleaning performance for all bristle types and brushing motions improved over the test period.

**Conclusion:**

Softer bristles consistently outperformed harder ones, indicating potential benefits for patients with orthodontic appliances. While horizontal brushing was more effective than circular motions, the direct application to clinical settings is limited due to the complexity of in-vivo toothbrush wear and individual brushing habits. Nonetheless, the choice of softer bristles and horizontal brushing techniques may increase the cleaning efficacy on orthodontic appliances.

For preventing oral diseases such as caries, gingivitis, and periodontitis the regular mechanical removal of biofilm from the teeth is essential.^1,2^ Patients with fixed orthodontic appliances face challenges with plaque biofilm removal, where higher levels of plaque retention have been reported, inducing dysbiosis and the formation of white spot lesions.^8,26,27^ As a result, a common issue after removing fixed dental appliances is orthodontically induced white spot lesions, which are hard to manage.^11,13,24^

A toothbrush in combination with toothpaste containing fluoride remains the most widely used and effective approach for removing plaque biofilm.^7,12^ Due to daily use, the toothbrush experiences bristle fatigue and bent filament tips, leading to a decrease in cleaning efficacy.^4,10,31^ However, extant research studies have generated differing results on effectiveness, indicating the need for further investigations.^6,29,30,36^

Lasance et al^18^ investigated the effects of manual toothbrush use on fixed orthodontic appliances in their study “Interplay Between the In-Vitro Cleaning Performance and Wear of Manual Toothbrushes in Fixed Orthodontic Appliances”. It is assumed that the toothbrushes undergo increased wear because the fixed appliances exert greater stress on the bristles. The study demonstrated an increase in cleaning efficacy with increased toothbrush wear in such a setting. One possible explanation is that the bristles become more flexible, enabling better adaptation to the contours of the tooth surface and improved cleaning of outer areas, including those around the bracket.^36^

The aim of this study was to investigate the influence of bristle hardness of manual toothbrushes on cleaning efficacy on teeth with fixed orthodontic appliances and their behavior during simulated aging of the toothbrushes. An in-vitro study was conducted using a soft toothbrush (with more flexible bristles) and a medium toothbrush (with harder bristles). To assess the cleaning efficacy, horizontal and circular brushing motions were performed.

The null hypothesis was that bristle hardness does not have an influence on their in-vitro cleaning performance in a typodont with fixed orthodontic appliances and this does not change during aging of the toothbrushes.

## MATERIALS AND METHODS

### Fabrication of Models and Toothbrushes

**Fig 1:**
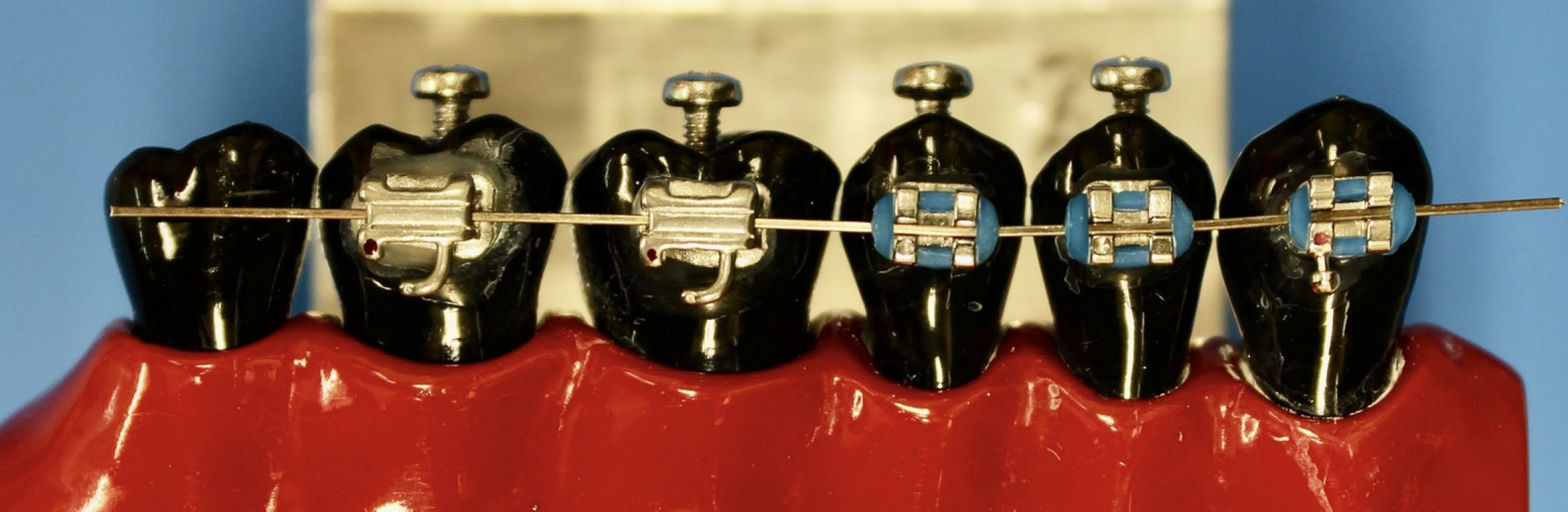
Teeth coated with black varnish inserted in typodont with brackets and wire.

An established method developed at the University of Zurich was used for measuring the cleaning efficacy of the toothbrushes.^18^ Therefore, typodonts of the second quadrant were used with black resin teeth (in-house production, University of Zürich, Switzerland). Polyurethane (Siladent; Goslar, Germany) was cast using a silicone mold based on the morphology of Frasaco plastic teeth (Frasaco; Tettnang, Germany). The whole posterior tooth segment was included containing teeth FDI 23 (canine), 24 and 25 (premolars), and 26 to 28 (molars) (Fig 1). Only teeth 24 to 26 were analyzed in subsequent stages because they provided a reproducible measurement area and could be brushed consistently by the brushing machine across the entire region of interest.

Using a bracket positioning gauge (Forestadent; Pforzheim, Germany) on the buccal surfaces of teeth 23, 24, and 25, brackets (Ormco; Glendora, CA, USA) were placed, while to the buccal surfaces of teeth 26 and 27 tubes (3M Oral Care; St Paul, MN, USA) were fixed. On the buccal surface, just before bonding the brackets and tubes to the teeth, a 2 x 3 mm area was roughened using a diamond drill (Jota; Rüthi, Switzerland).

A bonding agent (Heliobond, Ivoclar Vivadent; Opfikon, Switzerland) was applied and light-cured for 20 s. A single-component bonding agent (Monobond Plus, Ivoclar Vivadent) was applied to the bonding surface of the brackets and tubes and left to dry for 1 min. Subsequently, Heliobond (Ivoclar Vivadent) was applied to the bracket and tube bases and light cured for 20 s. For bonding the brackets and tubes to the teeth, a dual-curing composite (Variolink, Ivoclar Vivadent) was used, which was light cured for 20 s. A rectangular 0.016” x 0.022” stainless steel wire (G&H Orthodontics; Franklin, IN, USA) was placed into the brackets and tubes and affixed with elastic rubber ligatures (G&H Orthodontics).

Two types of toothbrushes, both of similar construction, were used. They featured a flat and parallel bristle field organized in three rows of nine individual filament bundles, resulting in a total of 27 bundles. The only difference between the two groups was their bristle hardness, which was achieved by varying the bristle length. The softer brush, Paro S27L (Esro; Kilchberg, Switzerland), had a bristle length of 11.5 mm, while the harder brush, Paro M27L (Esro; Kilchberg, Switzerland), featured a bristle length of 10 mm. Six toothbrush heads were glued (Loctite 480, Henkel; Düsseldorf, Germany) to clampable aluminum rods (in-house production, University of Zürich, Switzerland), making them usable in the brushing machines ZMB2 and ZMB8 (both in-house production, University of Zürich, Switzerland).

### Test Procedure

**Fig 2:**
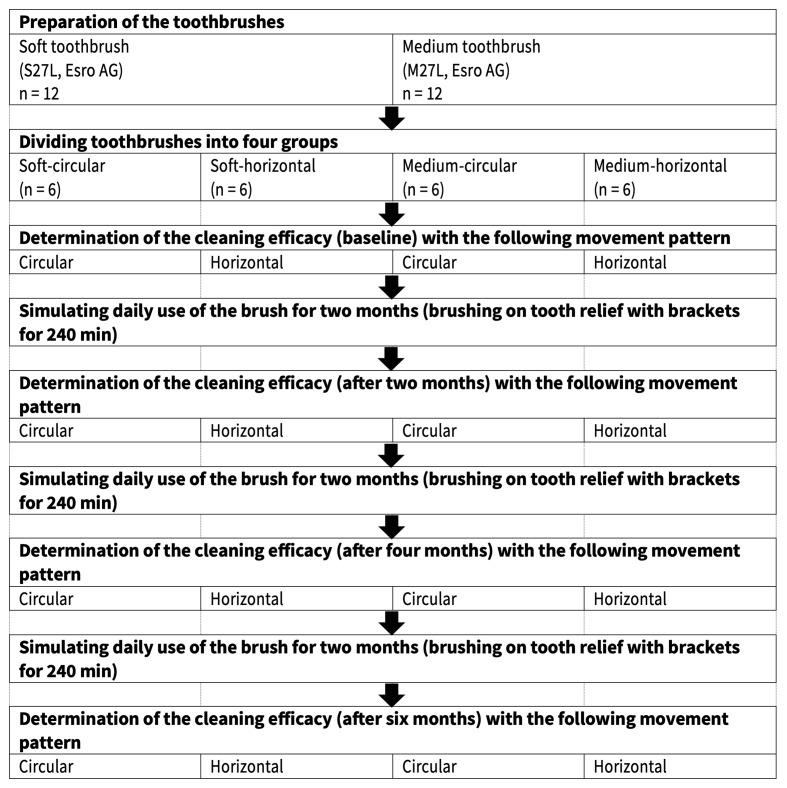
Visualization of the test procedure.

The cleaning efficacy was measured on three typodonts. On each model, two toothbrushes were used: toothbrushes 1 and 2 on typodont A, toothbrushes 3 and 4 on typodont B, and toothbrushes 5 and 6 on typodont C. The first evaluation run, at time point 0, was performed for the baseline measurement. For simulating the aging process of the toothbrush, they brushed over a 3D-printed tooth relief with brackets and a wire mounted on it. Aging was simulated in two-month cycles. The cleaning efficacy was measured after 2, 4, and 6 months of simulated toothbrush aging. The test procedure is illustrated in Fig 2.

### Determination of the Cleaning Efficacy

A white layer of titanium dioxide coat was applied to each tooth before each brushing procedure to assess the cleaning efficacy of the toothbrushes. The titanium dioxide coat contained 26% ethanol solution (Reuss-Chemie; Tägerig, Switzerland) mixed with titanium dioxide powder (Merck; Darmstadt, Germany) at a ratio of 2:1. According to previous studies, teeth 24 to 26 were hand coated with the titanium dioxide mixture using a brush.^3,32,33,36^ The coated teeth were left for 30 min to let the ethanol solvent evaporate.

The ZMB2 brushing machine was used for repeated, even brushing. The aluminum rods were clamped on the machine so that the center of the toothbrush heads glued to them was aligned with the center of the typodont underneath. With a spring balance (Pesola; Schindellegi, Switzerland), the contact pressure on the model of 2.5 N was determined and accordingly adjusted. Either a horizontal or circular brushing motion was performed. In horizontal brushing, the teeth were brushed back and forth at a frequency of 60 cycles/min. In circular brushing, a lateral motion of 60 cycles/min was added to create the movement. No toothpaste was used in the brushing procedure. The titanium dioxide enables visualization of the contact areas of the toothbrush with the surface of the tooth, that is, all areas appearing black were identified as cleaned by the bristles.^14^

**Fig 3:**
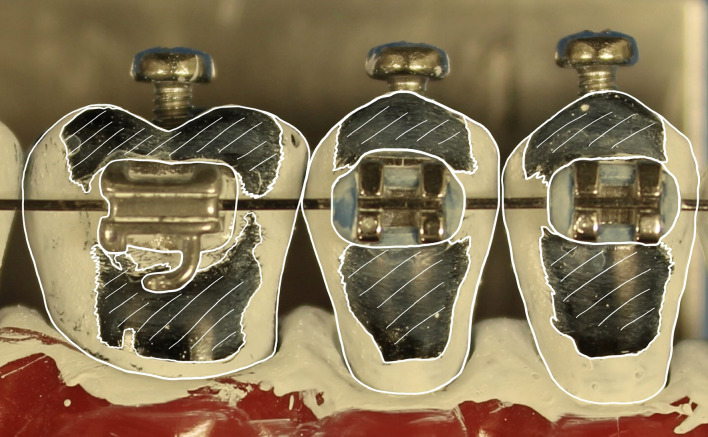
Masks of the three different areas that were measured: the entire tooth surface, the area of the bracket and all cleaned areas appearing black (hatched in white).

After a brushing cycle on a model, the amount of titanium dioxide removed from the surfaces of teeth 24 to 26 was quantified, enabling an assessment of the cleaning performance of the toothbrushes. To this end, the typodonts were photographed and analyzed with Fiji software for scientific image analysis (Fiji Team, https://fiji.sc/).^28^ To measure and calculate the area cleaned by the toothbrushes on the models, three different areas had to be measured: the entire tooth surface, the area of the bracket and all cleaned areas appearing black. A picture showing the respective areas on the teeth can be found in Fig 3. The area of the bracket was subtracted from the entire tooth surface, resulting in the overall area which needed to be cleaned. Then, the cleaned area was divided by the total area needing cleaning, given as a percentage. The percentage of cleaned tooth surface was calculated individually for the teeth 24, 25, and 26 and was combined to calculate the average of the three individual tooth values. This average represents the value for the cleaning efficacy for each individual model.

The models were cleaned with soap and water after every brushing session and set to dry for 30 min before being repainted and starting the next brushing process.

### Simulation of Usage of the Toothbrushes

**Fig 4:**
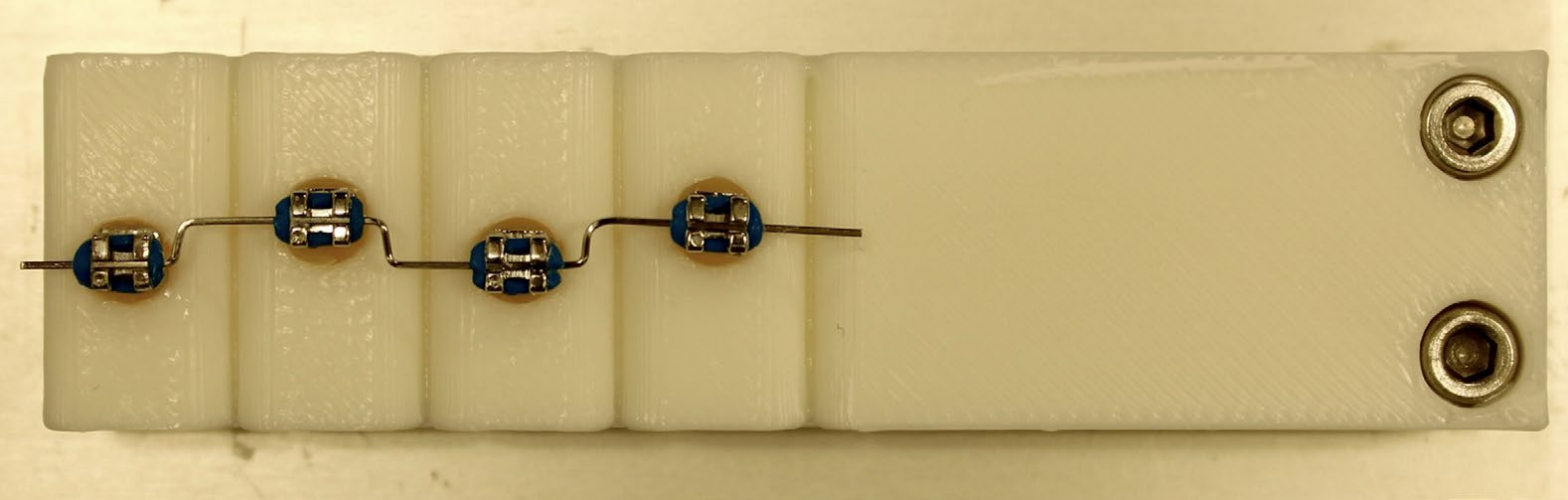
Simulated tooth relief featuring brackets and wire, designed for simulating ageing of the toothbrushes.

Bristle wear was simulated by brushing over a 3D-printed typodont mounted with brackets and wire. Made from a polylactide (PLA) plastic using a 3D printer (Bambu Lab X1 Carbon, Bambu Lab; Austin, TX, USA), the typodont simulated four adjacent teeth (waves). Brackets were placed 1 mm apart on the waves, alternating sides from the midline. This arrangement allowed simulation of wear along the width of the toothbrush. The brackets were attached using the same bonding procedure as bonding the brackets to the typodonts. The stainless-steel wire, brackets, and elastic rubber ligatures remained unchanged, as shown in Fig 4.

The toothbrushes were then clamped on the brushing machine ZMB8 to simulate wear, aligning them centrally on the tooth reliefs. With a spring balance, a contact pressure of 2.5 N was checked. Containers of toothpaste slurry were placed in the brushing machine so that the toothbrushes and tooth reliefs were fully submerged in slurry. Each container contained 75 g of toothpaste slurry freshly mixed just before the wear process was started. The slurry was prepared by mixing artificial saliva^15^ and Elmex toothpaste (GABA; Therwil, Switzerland) at a 2:1 ratio. The brushing machine moved the toothbrush heads over the tooth reliefs with back-and-forth movements at a frequency of 60 cycles/min. For an aging interval of two months, each usage simulation session lasted for 240 min. Therefore, to reach the full six months of aging it had to undergo a total of three aging cycles.

When the aging process was completed, the toothbrushes were rinsed with deionized water and left to dry. After drying, the toothbrushes were ready to undergo the next brushing cycle on the typodonts covered in titanium dioxide.

### Statistical Analysis

All statistical analyses and plots were computed with the statistical software R25, the packages tidyverse^34^ and betareg.^5^

In this two-by-two experimental setup, the effect of brushing motions (circular or horizontal) and type of toothbrush (soft or medium) on cleaned tooth surface (area in percentage) was investigated. Data were recorded at different time points, expressed by the variable duration of simulated usage (0, 2, 4 or 6 months).

In the inferential analysis, the focus was on the starting time of the experiment (duration of simulated usage 0 months) and on the last time point (duration of simulated usage 6 months). Beta regressions were performed to test the effect of brushing motions and type of toothbrush on the cleaned tooth surface.

No severe violations of model assumptions were observed. As the terms in the regression became statistically significant, pair-wise comparisons were conducted to understand the magnitude of the effect of the interactions between brushing motions and type of toothbrush on cleaned tooth surface. For this reason, pairwise comparisons of all groups were performed.

The level of statistical significance was set at p < 0.05 and the p-values were adjusted for multiple comparisons following the Tukey method.

An AI language model (GPT-5, OpenAI, accessed Jan 2026) was used solely for final language editing.

## RESULTS

The values for the cleaned surface area for different toothbrush types and brushing motions after various durations of simulated toothbrush use are presented in Table 1.

**Table 1:** Median/IQR values of the cleaned surface [%] for different toothbrush types and brushing motions after various durations of simulated toothbrush usage.

Toothbrush type	Brushing motion	Duration of simulated usage (in months)			
		0	2	4	6
Soft	circular	39.56/6.96^A^	66.53/2.44	56.30/5.58	49.56/5.17^a^
	horizontal	76.06/1.36^B^	80.45/4.02	76.88/4.45	79.41/4.79^b^
Medium	circular	28.13/4.51^C^	30.80/7.94	32.07/3.41	31.12/1.20^c^
	horizontal	56.62/2.31^D^	68.99/4.17	70.19/5.98	69.65/2.10^d^
Within the respective durations of simulated usage (0 or 6 months), values that are not statistically significantly different are marked with the same letters (upper case letters for 0 months of simulated usage and lower-case letters for 6 months of simulated usage).					

### Cleaning Performance

#### At baseline

For both kinds of brushing motions (circular or horizontal), the use of the soft toothbrushes resulted in statistically significantly better cleaning (p < 0.0001, respectively) compared to brushing with medium toothbrushes. Both types of toothbrushes (soft or medium) showed statistically significantly better cleaning potential when applied with horizontal brushing movements (p < 0.0001).

#### After 6 months of simulated use

Again, for both kinds of brushing motions (circular or horizontal), the use of the soft toothbrushes resulted in statistically significantly better cleaning (p < 0.0001, respectively) compared with brushing with medium toothbrushes. Both types of toothbrushes (soft or medium) again showed statistically significantly better cleaning potential when applied with horizontal brushing movements (p < 0.0001, respectively).

Also, after simulated use of two and four months, a higher percentage of cleaned area was observed when brushing with soft toothbrushes (within the respective kind of brushing movement) and with horizontal brushing movement (within the respective type of toothbrush).

## DISCUSSION

This in-vitro study evaluated the influence of bristle hardness (soft and medium) in manual toothbrushes on cleaning efficacy for teeth with fixed orthodontic appliances, examining both circular and horizontal brushing movements and their performance under simulated aging conditions. Using established methods on typodonts equipped with fixed orthodontic appliances, the study simulated toothbrush use to assess cleaning effectiveness.^14^ The results show that both at baseline and after 6 months of simulated use, soft bristles achieved statistically significantly better cleaning performance than medium bristles and horizontal brushing performed better than circular brushing (all p < 0.0001).

The controlled in-vitro setup provides valuable insights into how toothbrush type (soft or medium) and brushing movement (circular or horizontal) as well as toothbrush wear affects cleaning performance for orthodontic patients. Standardized conditions enhance reproducibility, yet the applicability of findings to clinical settings remains limited. Artificial bristle wear simulates natural usage only approximately, as real-life factors such as oral microorganisms, food particles, toothpaste abrasiveness, and natural bristle aging contribute to in-vivo wear. Additionally, individual variables, such as brushing technique, pressure, duration, and habits, e.g., chewing on the toothbrush, impact bristle wear.^16,17,20^ In orthodontic cases, the unique configuration of brackets and wires also likely influences both cleaning effectiveness and bristle wear. This study examined only one type of fixed appliance, so other systems may exhibit different cleaning and wear characteristics. Further, the relative effectiveness of various brushing techniques for orthodontic patients is a matter of debate;^21,23^ here, we focused on horizontal and circular brushing, commonly used methods.^9,14,22^

Based on clinical studies, a contact pressure of 2.5 N was applied during testing.^12,35^ Brushing duration was simulated using typical brushing habits, assuming twice-daily brushing for two minutes, totalling 120 min per month. Usage times at different intervals (2, 4, and 6 months) were calculated by multiplying monthly brushing duration by the corresponding number of simulated months. The study utilized Paro S27L and M27L toothbrushes, both of which have a flat brush head.

This study employed a titanium dioxide coating as a plaque biofilm substitute, an approach that lacks standardization but has been validated in previous studies by Imfeld et al^14^ and others.^26,28,32^ Nonetheless, it is important to acknowledge this study’s limitations. The titanium dioxide plaque biofilm substitute does not fully replicate the mechanical properties (viscosity, water insolubility, and abrasion resistance characteristic of real plaque biofilm) of dental plaque, potentially limiting the direct transferability of these findings to clinical practice.

Toothpaste was excluded because it dissolves titanium dioxide even without toothbrush bristle contact. The absence of toothpaste in the study design may affect results, as toothpaste enhances plaque removal through its abrasiveness and chemical agents.^12,15^

Despite these limitations, this model effectively replicates contact areas between the toothbrush and typodont, providing an approximate representation of cleaned surfaces.

The null hypothesis has to be rejected, as bristle hardness influenced in-vitro cleaning performance. The soft toothbrush consistently outperformed the medium toothbrush at baseline and after simulated 6-month use, irrespective of brushing motion. This correlates with prior studies showing that softer bristles better conform to the contours of the tooth surface, particularly around brackets.^26,32,36^ Increased flexibility in softer bristles likely facilitates access to difficult areas, such as spaces between brackets and teeth, enhancing plaque biofilm removal.

A prevailing assumption is that toothbrush effectiveness declines as bristle wear becomes visible.^31^ For orthodontic patients, additional stress from fixed appliances is thought to accelerate the bristle wear.^18,36^ However, in the present study worn toothbrushes maintained or even increased their cleaning efficacy irrespective of the type of toothbrush (soft or medium) and the applied brushing movement (circular or horizontal). These findings align with previous studies using similar methods on teeth without orthodontic appliances, where cleaning performance increased as toothbrushes aged.^36^

A potential explanation is that bristle flexibility increases with use, allowing better adaptation to the contours of teeth and orthodontic appliances, as previously discussed by Zoller et al.^36^ However, once a certain wear threshold is reached, bristle fraying and reduced contact pressure (2.5 N) may compromise cleaning efficacy or may no longer lead to further improvements.

Horizontal brushing showed better cleaning efficacy than circular brushing, potentially due to the bristles’ contact pattern with the appliance. During horizontal brushing, the bristles may more easily reach areas adjacent to the wire, whereas circular brushing tends to skip over it, leaving a broader uncleaned zone around the wire.

The horizontal brushing technique is particularly relevant for patients undergoing orthodontic treatment. However, it is associated with potential adverse effects, such as the development of wedge-shaped cervical defects, especially when applied with excessive pressure or over extended periods.^19,35^ Moreover, soft bristles may provide closer contact with the tooth surface and thereby improve cleaning efficacy, but they also increase the risk of dental hard tissue abrasion. These aspects were not investigated in the present study but should be considered, particularly for orthodontic therapy, which extends over longer periods and may therefore increase the likelihood of such adverse effects.

Clinically, these findings are relevant for patients with fixed orthodontic appliances, who face higher risks for white spot lesions due to plaque biofilm accumulation.^13,24^ Using softer-bristled toothbrushes and brushing horizontally may mitigate this risk by enhancing plaque biofilm removal. However, it should be noted that improvement of white spot lesions does not depend on brushing alone, but also on effective biofilm control, adequate prophylaxis with fluoride, and dietary/drink control to reduce frequent cariogenic challenges.^2,13,24^ Nevertheless, the observed maintenance or even improvement in cleaning performance with wear implies that patients may not need to replace their toothbrushes as frequently,^18,36^ offering both economic and environmental benefits.

Future research should focus on long-term in-vivo studies to verify these findings under real-world conditions. Testing a variety of orthodontic appliances and toothbrush models could offer further insights into optimized oral hygiene strategies for orthodontic patients. Additionally, incorporating toothpaste in experimental setups could provide a more comprehensive understanding of its role in cleaning efficacy.

## CONCLUSION

Toothbrushes maintain or even improve cleaning performance over time. Softer bristles consistently outperformed harder ones, supporting their potential benefits for orthodontic patients. While horizontal brushing proved more effective than circular motions post-simulation, the direct clinical applicability is constrained by the complexities of in-vivo wear and individual brushing habits. Nonetheless, recommending softer bristles and horizontal brushing techniques may improve cleaning efficacy for orthodontic patients.

## ACKNOWLEDGMENTS

The study was conducted as the doctoral thesis of med. dent. Damian Wüthrich and performed at the Center for Dental Medicine of the University of Zürich, Switzerland, under the supervision of Prof. Dr. F. J. Wegehaupt and Prof. Dr. Dr. h.c. T. Attin. The authors declare no conflicts of interest.

## REFERENCES
